# Factors Affecting Repeatability of Assessment of the Retinal Microvasculature Using Optical Coherence Tomography Angiography in Healthy Subjects

**DOI:** 10.1038/s41598-019-52782-6

**Published:** 2019-11-08

**Authors:** Taek Hoon Lee, Hyung Bin Lim, Ki Yup Nam, Kyeungmin Kim, Jung Yeul Kim

**Affiliations:** 1 Rhee’s Eye Hospital, Daejeon, Republic of Korea; 2 Graduate School of Medicine, Chungnam National University College of Medicine, Daejeon, Republic of Korea; 3 Department of Ophthalmology, Chungnam National University College of Medicine, Daejeon, Republic of Korea; 4 Department of Ophthalmology, Gyeongsang National University Changwon Hospital, Changwon, Republic of Korea

**Keywords:** Medical imaging, Eye manifestations

## Abstract

Various factors can affect repeatability of optical coherence tomography angiography (OCTA) measurements, and they have not been studied sufficiently. We aimed to investigate the factors associated with the repeatability of automated superficial retinal vessel density (VD) and foveal avascular zone (FAZ) metrics acquired from OCTA. A total of 141 normal eyes from 141 healthy subjects were included, and two consecutive macular 6 × 6-mm angiography scans were performed. VD, perfusion density (PD), and FAZ of the superficial capillary plexus were calculated automatically. Reproducibility was assessed based on intraclass correlations (ICCs) and coefficients of variation (CVs). VD (ICC: 0.824, CV: 3.898) and PD (ICC: 0.845, CV: 4.042) over the entire 6-mm scan area showed better repeatability than VD (ICC: 0.752, CV: 17.470) and PD (ICC: 0.752, CV: 18.552) in the 1-mm scan, and with respect to the obtained FAZ metrics (ICC < 0.75, CV > 10.0%). Regression analyses showed that two factors, signal strength (p = 0.004) and average VD over the total 6-mm scan area (p < 0.001), were significantly correlated with the CV of the VD. Signal strength was associated with the repeatability of OCTA measurements and should be considered in the analysis of retinal VD and FAZ.

## Introduction

Optical coherence tomography angiography (OCTA) is a rapidly evolving technique that provides a fast, noninvasive assessment of retinal and choroidal microvascular flow^1^. OCTA can be used to differentiate and visualize the microvasculature of various retinal and choroidal layers at different depths, which is difficult to achieve using conventional fluorescein or indocyanine green angiography^2^. OCTA also allows for quantitative evaluation of retinal and choroidal blood flow using several vascular metrics, such as vessel density (VD), perfusion density (PD), and foveal avascular zone (FAZ)^3,4^. Numerous studies have reported that these vascular metrics may be useful for monitoring and detecting age-related macular degeneration^5^, diabetic retinopathy^6^, retinal vein occlusion^7^, and glaucoma^8^.

Confidence in blood flow metrics, as measured by OCTA during clinical assessments and for research purposes, requires demonstration of repeatability, particularly between subjects. In addition, OCTA measurements can be affected by several factors, including ocular anatomy, data acquisition methods, and image quality. Although several studies have demonstrated high repeatability of OCTA measurements, not only in normal eyes but also in those with disease^9–11^, the factors associated with repeatability have not been studied sufficiently. The ability to control these factors would allow clinicians to obtain more reliable blood flow metrics with OCTA evaluation.

Therefore, the purpose of the current study was to determine the repeatability of automatic OCTA measurements in healthy subjects by identifying the factors that have a significant effect on the repeatability of OCTA measurements.

## Results

### Demographics

This study initially included of 164 eyes from 164 subjects, and 23 eyes were excluded due to segmentation error or motion artifact. Finally, a total of 141 normal eyes from 83 men and 58 women (mean age, 46.1 ± 17.1 years) were examined in this study. The mean visual acuity of the subjects was −0.03 ± 0.08 logMAR, and the mean spherical equivalent and, intraocular pressure, and axial length were −1.32 ± 2.71 D, 15.4 ± 3.0 mmHg, and 24.2 ± 1.2 mm, respectively (Table 1).

**Table 1:** Demographics.

Eyes (n)	141
Age (mean ± SD, years)	46.1 ± 17.1
Sex (male/female)	83/58
BCVA (mean ± SD, logMAR)	−0.03 ± 0.08
Spherical equivalent (mean ± SD, diopters)	−1.32 ± 2.71
Intraocular pressure (mean ± SD, mmHg)	15.4 ± 3.0
Axial length (mean ± SD, mm)	24.2 ± 1.2
Lens status (n, %)	
Phakia	
Nucleosclerosis, Grade 0–1	128 (90.8)
Nucelosclerosis, Grade 2	5 (3.5)
Pseudophakia	8 (5.7)

SD, standard deviation; BCVA, best-corrected visual acuity; logMAR, logarithm of the minimum angle of resolution.

### Repeatability of OCTA measurements

There was no statistically significant difference in the VD, PD, or FAZ values obtained by two consecutive 6-mm scans performed with a 5-min interval (Table 2). ICC values achieved a better than ‘good’ grade for all parameters, but differed among measurement areas. VD and PD showed excellent reproducibility, while FAZ was classified as good. The CV was less than 10% for VD and PD for the 6-mm scan of the entire area; however, CV > 10% for VD and PD for 1-mm scans and the FAZ.

**Table 2:** The repeatability of two consecutive angiography measurements: vascular density, perfusion density, and foveal avascular zone metrics.

	First value	Second value	p-value	ICC (95% CI)	CV (95% CI)
**VD**					
6-mm (entire area; mean ± SD, mm^−1^)	17.562 ± 1.879	17.684 ± 1.662	0.354	0.824 (0.745–0.881)	3.898 (3.111–4.685)
Outer ring (mean ± SD, mm^−1^)	17.563 ± 1.74	17.652 ± 1.557	0.355	0.882 (0.836–0.916)	3.512 (2.811–4.213)
Inner ring (mean ± SD, mm^−1^)	16.974 ± 2.595	17.171 ± 2.216	0.191	0.859 (0.801–0.898)	5.943 (4.597–7.288)
1-mm (center; mean ± SD, mm^−1^)	8.718 ± 3.374	8.816 ± 3.088	0.126	0.752 (0.701–0.822)	17.470 (14.120–20.820)
**PD**					
6-mm (entire area; mean ± SD)	0.419 ± 0.050	0.432 ± 0.045	0.287	0.845 (0.772–0.891)	4.042 (3.198–4.887)
Outer ring (mean ± SD, mm^−1^)	0.433 ± 0.047	0.435 ± 0.043	0.310	0.867 (0.814–0.904)	3.661 (2.895–4.427)
Inner ring (mean ± SD, mm^−1^)	0.402 ± 0.066	0.408 ± 0.058	0.157	0.843 (0.782–0.888)	6.295 (4.865–7.725)
1-mm (center; mean ± SD)	0.181 ± 0.079	0.197 ± 0.074	0.906	0.752 (0.674–0.772)	18.552 (15.079–22.025)
**FAZ**					
Entire area (mean ± SD, mm^2^)	0.267 ± 0.126	0.278 ± 0.141	0.314	0.705 (0.645–0.830)	18.166 (14.263–22.068)
Perimeter (mean ± SD, mm)	2.155 ± 0.644	2.148 ± 0.668	0.648	0.685 (0.554–0.775)	11.926 (9.119–14.733)
Circularity (mean ± SD)	0.707 ± 0.130	0.720 ± 0.110	0.194	0.603 (0.384–0.770)	10.966 (8.539–13.392)

ICC = intraclass coefficient; CV = coefficient of variation; CI = confidence interval; VD = vessel density; PD = perfusion density; FAZ = foveal avascular zone.

### Correlation analyses: factors associated with OCTA measurement repeatability

Pearson’s correlation was used to analyze the relationship between various factors and the repeatability of VD OCTA measurements in 6-mm scans (Table 3). Age, BCVA, IOP, spherical equivalent, axial length, and CMT were not significantly associated with the absolute differences or CVs of the two 6-mm scans (all p > 0.05). The VD of the 6-mm scan showed a significant negative correlation with the absolute differences and CVs of VD and PD for the total scan area (all p < 0.001), and were also significantly correlated with the CV of the FAZ (r = −0.326, p < 0.001). In addition, signal strength showed statistically significant negative correlations with absolute differences and CVs of VD, PD, and FAZ (all p < 0.01), indicating that the higher the signal strength, the better the repeatability. In multivariate linear regression analyses, signal strength (partial r = 0.246, p = 0.004) and VD from the 6-mm scans (partial r = −0.577, p < 0.001) showed a significant association with the CV of VD (Table 4).

**Table 3:** Pearson’s correlation analysis results of various clinical factors and repeatability parameters of optical coherence tomography angiography measurements

	VD, 6-mm area				PD, 6-mm area				FAZ			
	Absolute difference		CV		Absolute difference		CV		Absolute difference		CV	
	r	p-value	r	p-value	r	p-value	R	p-value	r	p-value	r	p-value
Age	−0.132	0.118	−0.172	0.058	−0.153	0.070	−0.159	0.078	0.166	0.070	0.053	0.538
BCVA (logMAR)	0.090	0.349	0.108	0.260	0.057	0.553	0.080	0.402	0.050	0.605	0.104	0.277
IOP	−0.049	0.616	−0.051	0.597	−0.053	0.583	−0.054	0.577	0.005	0.961	0.033	0.733
Spherical equivalent	0.111	0.249	0.122	0.208	0.099	0.306	0.107	0.266	0.155	0.108	0.162	0.094
Axial length	−0.043	0.661	−0.055	0.570	−0.047	0.626	−0.058	0.548	−0.116	0.234	−0.133	0.169
CMT	0.082	0.336	0.076	0.368	0.081	0.337	0.078	0.355	−0.154	0.068	−0.005	0.950
VD, 1-mm area^*^	**−0**.**310**	**<0**.**001**	**−0**.**352**	**<0**.**001**	**−0**.**308**	**<0**.**001**	**−0**.**350**	**<0**.**001**	**−0**.**296**	**<0**.**001**	**−0**.**221**	**0**.**009**
VD, 6-mm area^*^	**−0**.**540**	**<0**.**001**	**−0**.**631**	**<0**.**001**	**−0**.**533**	**<0**.**001**	**−0**.**628**	**<0**.**001**	−0.115	0.173	**−0**.**326**	**<0**.**001**
FAZ^*^	−0.141	0.099	**−0**.**172**	**0**.**044**	−0.137	0.109	**−0**.**173**	**0**.**043**	**0**.**336**	**<0**.**001**	−0.137	0.111
Signal strength^*^	**−0**.**331**	**<0**.**001**	**−0**.**384**	**<0**.**001**	**−0**.**335**	**<0**.**001**	**−0**.**393**	**<0**.**001**	**−0**.**281**	**0**.**002**	**−0**.**263**	**0**.**007**

VD = vessel density; PD = perfusion density; FAZ = foveal avascular zone; BCVA = best-corrected visual acuity; IOP = intraocular pressure; CMT = central macular thickness; CV = coefficient of variation.

^*^The average values in two consecutive angiography scans were subjected to correlation analysis.

Boldface numbers indicate statistically significant differences at p < 0.05.

**Table 4:** Multivariate linear regression analysis of significant factors in Pearson’s correlation analysis and repeatability parameters of vessel density for the 6-mm scan.

	B	Partial r	p-value
Signal strength^*^	1.917 ± 0.653	0.246	**0**.**004**
VD, 6-mm area^*^	−2.531 ± 0.309	−0.577	**<0**.**001**
FAZ^*^	−3.446 ± 2.672	−0.111	0.199

VD = vessel density; FAZ = foveal avascular zone.

^*^The average values in two consecutive angiography scans were used for correlation analysis.

Boldface numbers indicate statistically significant differences at p < 0.05.

### Effect of signal strength

To confirm the effect of signal strength, we compared the OCTA measurements of 25 subjects for whom signal strength differed between the two consecutive scans (signal strength values of 8 and 9). Significant differences were observed in the measured parameters between the two signal strengths, with the exception of FAZ (Table 5).

**Table 5:** Comparison of vascular density, perfusion density, and foveal avascular zone metrics between two consecutive macular angiography scans (n = 25).

	First measurement (signal strength = 8)	Second measurement (signal strength = 9)	p-value
**VD**			
6-mm (entire area; mean ± SD, mm^−1^)	15.33 ± 1.93	16.70 ± 1.33	**<0**.**001**
1-mm (center; mean ± SD, mm^−1^)	6.70 ± 3.25	8.11 ± 2.96	**0**.**001**
**PD**			
6-mm (entire area; mean ± SD)	0.37 ± 0.05	0.40 ± 0.04	**<0**.**001**
1-mm (center; mean ± SD)	0.14 ± 0.08	0.18 ± 0.07	**0**.**002**
**FAZ**			
Entire area (mean ± SD, mm^2^)	0.18 ± 0.09	0.22 ± 0.10	**0**.**043**
Perimeter (mean ± SD, mm)	1.79 ± 0.43	1.84 ± 0.46	0.833
Circularity (mean ± SD)	0.68 ± 0.09	0.74 ± 0.08	**0**.**013**

VD = vessel density; PD = perfusion density; FAZ = foveal avascular zone; SD = standard deviation.

The p-value was obtained using a Wilcoxon signed rank test.

Boldface numbers indicate statistically significant differences at p < 0.05.

**Figure 1:**
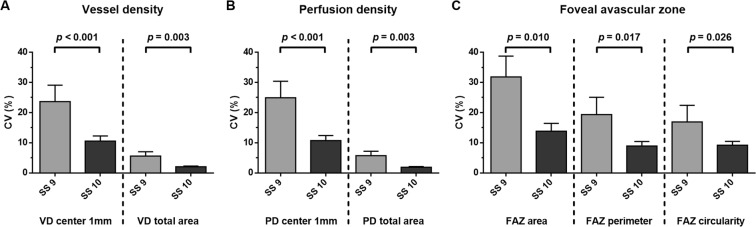
Comparison of the coefficients of variation of subjects showing the same signal strength across two scans, of 9 (n = 25) or 10 (n = 63). All parameters of the signal strength 10 group, including VD, PD, and FAZ metrics, showed better repeatability than those of the signal strength 9 group (all p < 0.05).

To investigate the effect of signal strength on measurement reproducibility, we compared the CV values of subjects for whom signal strength was the same for the two consecutive scans, with both having a value of either 9 (n = 25) or 10 (n = 63). Both the VD and PD values for the 6-mm scan, and FAZ metrics, showed lower CV values in the group with a signal strength of 10 for both scans (Fig. 1).

## Discussion

In previous studies, OCTA repeatability and reproducibility were generally good in both normal and diseased eyes. You *et al*. analyzed the intra- and inter-visit reproducibility of vascular density of superficial macula under various conditions [analysis range, retina state (normal or not), etc.] in normal patients and patients with retinal disease^12^. Although the repeatability and reproducibility were better in the normal group than in those with retinal disease, the CV value was <5% in most cases and showed good repeatability in both groups.

Lei *et al*. also analyzed the repeatability and reproducibility of measurements of the vascular density and PD of the superficial macula in normal and retinal disease eyes^11^. They performed three consecutive measurements using the same OCTA machine and analyzed the repeatability and reproducibility. The CV was less than 6% for both VD and PD repeatability and reproducibility, under various conditions. The ICC was high for both repeatability (0.82–0.98) and reproducibility (0.62–0.95).

In an earlier study, we analyzed the repeatability of OCTA VD measurements of the superficial macula in 134 patients with various retinal diseases that could cause deformation of the retinal structure^9^. Although previous studies have reported that pigment epithelium detachment and/or drusen interfere with the accuracy of VD measurements, our previous study of retinal disease cases showed good reproducibility, where the ICC value ranged from 0.737–0.905 and CV from 5.60–8.86% in^13^.

**Figure 2:**
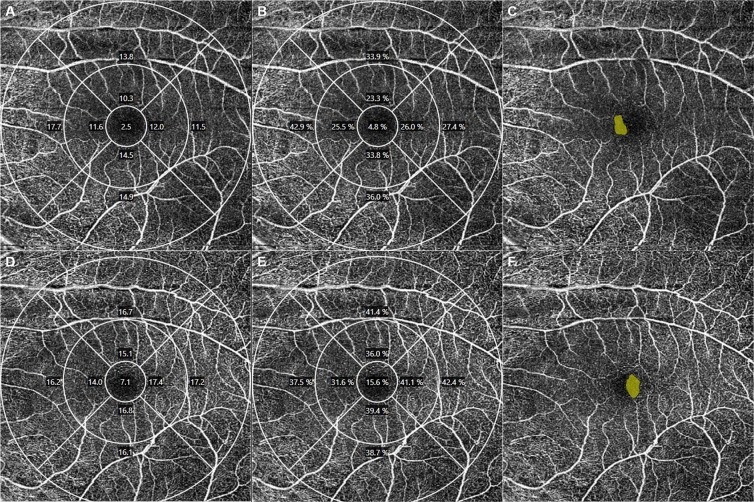
Optical coherence tomography angiography (OCTA) images of a 60-year-old male subject with nuclear sclerosis grade 1. Signal strength of the upper and lower row images which scanned consecutively was 8, and both OCTA images showed significant difference values [vessel density in the 6 mm full area: 13.5 mm^−1^ (**A**) vs. 16.1 mm^−1^ (**D**); perfusion density in the 6 mm full area: 0.322 (**B**) vs. 0.386 (**E**); foveal avascular zone area: 0.10 mm^2^ (**C**) vs. 0.13 mm^2^ (**F**)].

**Figure 3:**
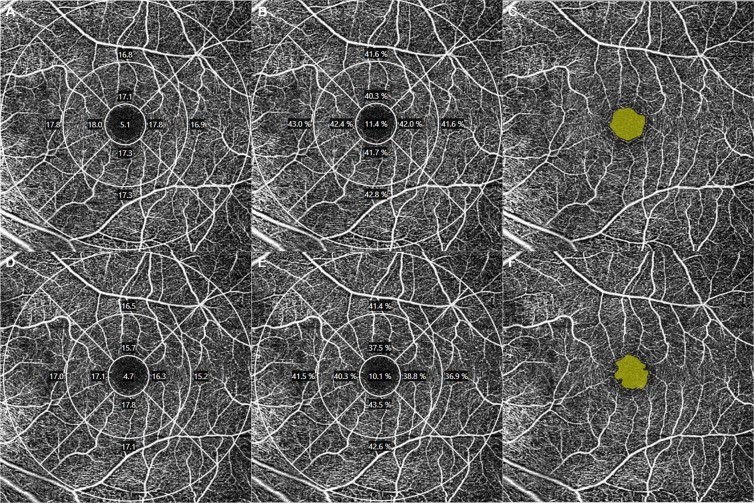
Optical coherence tomography angiography (OCTA) images of a 68-year-old male subject with pseudophakia. Signal strength of the upper and lower row images which scanned consecutively was 10, and both OCTA images showed similar values [vessel density in the 6 mm full area: 16.2 mm^−1^ (**A**) vs. 16.9 mm^−1^ (**D**), perfusion density in the 6 mm full area: 0.396 (**B**) vs. 0.412 (**E**); foveal avascular zone area: 0.45 mm^2^ (**C**) vs. 0.46 mm^2^ (**F**)].

Despite the good repeatability and reproducibility of OCTA measurements in normal and diseased eyes reported in many studies, certain factors influence the readings, including the scan location, scan area (3 × 3-mm vs. 6 × 6-mm scans), signal strength, and retinal abnormalities. Therefore, we investigated factors that might affect the repeatability of OCTA measurements. Unlike previous studies, we analyzed OCTA results for normal eyes, and found that the signal strength had significant negative correlations with the absolute difference and CV of two measurements of VD and PD, based on 6-mm scans, and the FAZ. In other words, the higher the signal strength, the lower the CV value, and the better the repeatability (Figs 2 and 3). These results are consistent with those of Lei *et al*. showing that signal strength is a significant factor in the reproducibility of both VD and PD readings^11^. Signal strength showed a strong correlation with VD and PD in one of our previous studies^14^. In addition, Yu *et al*. reported that signal strength reduction which was induced by beam attenuation and defocus significantly affected OCTA measurements, and noted that signal strength should be considered in the interpretation of OCTA measurements^15^.

In this study, during the calculation of the VD and PD, repeated B-scans were acquired from the same tissue location, and motion contrast was created by calculating the decorrelation of repeated B-scans. A thresholding algorithm was applied to the obtained unthresholded OCTA B-scans to remove invalid pixels associated with low or noisy OCTA pixels, and thus yield a thresholded OCT-B scan image^16^. During this process, the size of the vessel is an important factor, as large vessels are less affected by signal strength; however, signals from small vessels may be attenuated or undetected^17^. The macula has only small vessels, and signal strength therein thus correlates strongly with VD and PD reproducibility.

In this study, reproducibility indexed by ICC and CV for normal subjects was generally good; however, low reproducibility was also seen occasionally. The ICC values indicated excellent reproducibility in terms of VD and PD, but FAZ achieved only a ‘good’ classification. The CV values of VD and PD for the 6-mm scans showed good reproducibility (<10%). However, with regard to the PD in 1-mm scans of the central foveal area and the FAZ, the CV values exceeded 10%. In the report of You *et al*.^12^, superficial VD reproducibility was found to be lower in the central foveal area, because the retinal vessels are located only in the periphery of the central measurement area and the size and shape of the FAZ varies from person to person. This is similar to the results in the current study.

We suggest that if this study had used 3 × 3-mm OCTA scans, higher reproducibility of VD and PD values of the superficial macula would have been obtained. The 3 × 3-mm scan has better resolution than the 6 × 6-mm scan, with a scan interval of 12.2 µm compared to 17.1 µm^4^. This difference in scan density is expected to improve the repeatability of 3 × 3-mm OCTA scans. In addition, 3 × 3-mm scans measure approximately 60,000 points, while 6 × 6-mm scans measure approximately 120,000 points; as such, the 6 × 6-mm scan takes about twice as long, rendering it more susceptible to motion artifacts.

This study had the limitation of a retrospective, observational design. Previous studies included only a small number of patients, despite being concerned with both normal and diseased eyes. As our study included only normal patients, the results are meaningful in that the assessment of the reproducibility of OCTA was not affected by structural changes in the retina. Second, because 6 × 6 mm scan was used for analysis in this study, we could not confirm how the SS affected the analyses of other areas, such as the optic disc, or other scan protocols, such as the 3 × 3 mm angiography scans. Third, we included subjects with mild cataract in this study. However, mild cataracts can also affect image quality. Finally, Li *et al*.^18^. reported that the elongation of axial length lowered the repeatability of OCTA measurements, whereas no significant association between axial length and repeatability was found in our study. We excluded subjects with high myopia from this study and could not identify any trends of OCTA repeatability in the long eye. This should be clarified in future studies.

In conclusion, signal strength significantly affected OCTA repeatability in this study. Therefore, signal strength should be a key consideration in OCTA analyses of the retinal microvasculature and FAZ, where it is necessary to achieve the highest possible signal strength.

## Methods

### Subjects

This was a retrospective observational study. Patient medical data recorded at the Retina Clinic of Chungnam National University Hospital (Daejeon, Republic of Korea) were evaluated. No enrolled patient had participated in any other studies. The study protocol was approved by the institutional review board of Chungnam National University Hospital and adhered to the tenets of the Declaration of Helsinki. The requirement for obtaining informed patient consent was waived due to the retrospective nature of the study.

Among the healthy normal patients who visited our clinic for various reasons (health screening checkups, workup for ocular disease, etc.), those who met the study inclusion and exclusion criteria were included. All subjects underwent comprehensive ophthalmic examinations including a slit-lamp examination, best-corrected visual acuity (BCVA), intraocular pressure (IOP) measurements, dilated fundus examination, fundus photography, measurement of axial length using the IOLMaster^®^ (Carl Zeiss Meditec, Jena, Germany), and of OCT and OCTA using the Zeiss Cirrus 5000 system (Carl Zeiss Meditec, Dublin, CA, USA). The inclusion criteria included age of 20–79 years, BCVA > 20/25 (Snellen), spherical equivalent within ±6 diopters (D), high-quality fundus image, absence of glaucomatous and other optic neuropathies, absence of retinal nerve fiber layer defects based on fundus photography, and no previous intraocular surgeries or procedures. Exclusion criteria included the presence of diabetes or hypertension, a history of retinal neuro-ophthalmic disease and glaucoma, a history of ocular trauma, BCVA < 20/25, IOP > 21 mmHg, spherical equivalent >  + 6.0 D or < −6.0 D, axial length ≥ 26.0 mm, and the presence of segmentation errors or motion artifacts in the angiography scan. Considering the possibility of deterioration of image quality, nuclear sclerosis (according to the Lens Opacity Classification System III^19^) grade 3 and above were excluded from the study. If both eyes met the inclusion criteria, one eye was randomly selected.

### OCTA

The Zeiss Cirrus HD-OCT 5000 with AngioPlex^™^ (Carl Zeiss Meditec) was used to acquire microvasculature images of macular areas. All eyes underwent macular angiography imaging (two 6 × 6-mm scans at 5-min intervals) with the pupils dilated. The Zeiss Cirrus instrument operated at a central wavelength of 840 nm and a speed of 68,000 A-scans per second. There were 350 A-scans in each B-scan along the horizontal and vertical dimensions^4^. The Optical Micro Angiography Complex (OMAG^c^) was used to analyze changes in intensity and phase within the sequential B-scans performed at the same position^20,21^, to produce en face microvascular images. The vascular images of the superficial capillary plexus, which spanned the region from the internal limiting membrane to the inner plexiform layer, and the deep capillary plexus, which extended from the inner nuclear layer to the outer plexiform layer, were displayed automatically. The AngioPlex^™^ system incorporates FastTrac^™^ retinal-tracking technology to minimize motion artifacts.

All scans were analyzed using Cirrus OCTA software (AngioPlex^™^, version 10.0). The measurement area of the 6 × 6-mm scan was divided into nine subfields according to the Early Treatment of Diabetic Retinopathy study. VD (defined as the total length of the perfused vasculature per unit area in the region of measurement) and PD (defined as the total area of perfused vasculature per unit area in the region of measurement) of each subfield were measured automatically, as well as the FAZ area, perimeter, and circularity (defined as 4πA/P, where *A* and *P* represent the area and perimeter, respectively)^22^. All OCTA scans were performed by the same experienced examiner, and all scans were reviewed individually by two investigators, for loss of fixation, segmentation errors, and motion artifacts; substandard scans were excluded. The central macular thickness (CMT) was also measured using a 512 × 128 macular cube combination scan mode.

### Repeatability assessment

Repeatability was assessed based on intraclass correlations (ICCs) and coefficients of variation (CV). The within-subject standard deviation (Sw) was calculated as the square root of the within-subject variance^23^. CV was calculated as CV = Sw/average of the measurements) × 100; CV < 10% indicated good reproducibility. The ICC is a statistical parameter that summarizes the reproducibility of a given group of subjects. It is based on variance component analyses and indicates the variance attributable to real differences between subjects as a proportion of the total variation^24^. ICC values were classified as follows: poor (ICC < 0.40), fair (0.40 ≤ ICC < 0.60), good (0.60 ≤ ICC < 0.75), or excellent (ICC ≥ 0.75)^25^.

### Statistical analyses

The VD, PD, and FAZ for each of two consecutive scans were compared using the paired *t*-test, and the ICC and CV values were calculated. To identify factors that affect OCTA measurement repeatability, Pearson’s correlation and linear regression analyses of the absolute difference and CV of the VD value of the two scans (for the 6-mm scans of the entire area) were performed.

All statistical analyses were conducted using SPSS statistical software for Windows (version 18.0; SPSS Inc., Chicago, IL, USA) and GraphPad Prism^TM^ (version 6.0; GraphPad Software, San Diego, CA, USA). Snellen BCVA results were converted into the logarithm of the minimum angle of resolution value (logMAR). Continuous variables are presented as the mean ± standard deviation. Differences were considered significant at p < 0.05.

## Data Availability

Data supporting the findings of the current study are available from the corresponding author on reasonable request.
