# Publisher Correction: Factors Affecting Repeatability of Assessment of the Retinal Microvasculature Using Optical Coherence Tomography Angiography in Healthy Subjects

**DOI:** 10.1038/s41598-020-61263-0

**Published:** 2020-03-11

**Authors:** Taek Hoon Lee, Hyung Bin Lim, Ki Yup Nam, Kyeungmin Kim, Jung Yeul Kim

**Affiliations:** 1 Rhee’s Eye Hospital, Daejeon, Republic of Korea; 2 Graduate School of Medicine, Chungnam National University College of Medicine, Daejeon, Republic of Korea; 3 Department of Ophthalmology, Chungnam National University College of Medicine, Daejeon, Republic of Korea; 4 Department of Ophthalmology, Gyeongsang National University Changwon Hospital, Changwon, Republic of Korea

Correction to: *Scientific Reports* 10.1038/s41598-019-52782-6, published online 08 November 2019

In the original version of this Article, the author Hyung Bin Lim was incorrectly indexed. This error has now been corrected.

